# Application of CRISPR/CasΦ2 System for Genome Editing in Plants

**DOI:** 10.3390/ijms23105755

**Published:** 2022-05-20

**Authors:** Qinan Cai, Dongmei Guo, Yujun Cao, Yuan Li, Rui Ma, Wenping Liu

**Affiliations:** 1 Maize Resources Institute, Jilin Academy of Agricultural Sciences, Gongzhuling 136100, China; qinancai@126.com; 2 Key Laboratory of Agricultural Biotechnology, Jilin Academy of Agricultural Sciences, Changchun 130033, China; l147856329@163.com; 3 Institute of Agro-Product Safety and Nutrition, Zhejiang Academy of Agricultural Sciences, Hangzhou 310021, China; gdm100200@163.com; 4 Institute of Agricultural Resources and Environment, Jilin Academy of Agricultural Sciences, Changchun 130033, China; caoyujun828@163.com

**Keywords:** CRISPR, CasΦ2, genome editing, Mg^2+^, *Arabidopsis thaliana*, *Nicotiana benthamiana*

## Abstract

CRISPR/Cas system has developed a new technology to modify target genes. In this study, CasΦ2 is a newly Cas protein that we used for genome modification in Arabidopsis and tobacco. *PDS* and *BRI1* of marker genes were chosen for targeting. CasΦ2 has the function to cleave pre-crRNA. In the presence of 10 mM Mg^2+^ irons concentration, sgRNA3 type guided CasΦ2 to edit target gene and generate mutation, and a mutant seedling of *AtBRI1* gene with an expected male sterile phenotype was obtained. In the process of tobacco transformation, the gene editing activity of CasΦ2 can be activated by 100 nM Mg^2+^ irons concentration, and sgRNA1 type guided CasΦ2 to edit target gene. Mutant seedlings of *NtPDS* gene with an expected albino were obtained. The results indicate that CasΦ2 can effectively edit target genes under the guidance of different sgRNA type in the presence of Mg^2+^ ions. Together, our results verify that the CRISPR/CasΦ2 system is an effective and precise tool for genome editing in plants.

## 1. Introduction

In crop germplasm resources, rare mutations can lead to qualitative changes in traits and increase crop yield. However, these mutations are extremely hard to obtain in natural populations [1]. The target genes can be modified precisely and accurately by a genome editing system, and the desired mutation can be produced, which makes it possible to introduce the improved gene directly into the elite varieties [2,3]. Over the last decade, the clustered regularly interspaced short palindromic repeat/CRISPR-associated protein (CRISPR/Cas) systems have become one of the main methods of gene modification in animals and plants. Cas protein can generate double-strand breaks of DNA at the location of target site sequence on the genome under the guidance of RNA. DNA repair is usually accomplished by non-homologous end-joining (NHEJ) and homologous-directed repair (HDR). The NJHE pathway is often error-prone and generates indels in the repair process. Therefore, the desired mutation can be generated at the desired target gene sites through CRISPR/Cas systems [4].

The CRISPR/Cas systems include two general classes, Class I and Class II. According to their composition, they can be further divided into 6 types and 33 subtypes. In particular, *Cas* genes in Class II have been extensively studied [5,6]. In 2013, *Cas9* gene to be edit target genes was reported in mammalian cells [7]. However, SpCas9 recognizes protospacer adjacent motif (PAM) bases limitation behind target site sequence, and SpCas9 could not edit some target sequences in the genome. So, some key amino acids of SpCas9 have been mutated to generate Cas9-NG, xCas9 and SpRY, respectively, which broaden the recognized PAM and allow more target sites to be edited in the genome [8,9,10,11]. Cas9 variants have broadened PAM requirements and have allowed for gene editing at more target sites in the genome to attain research requirements compared with Cas9 [12]. Since then, many *Cas* genes for plant genome editing have been reported. The second most widely used Cas protein in plant genome editing is Cas12 [13,14]. Subsequently, *Cas13* and *Cas14* were discovered and applied to plant genome editing [15,16]. These Cas proteins can modify precise and efficient target sites using insertions, deletions and substitutions. They can be combined with other editors to form different editing systems, which broadens their editing function and promoters the research of plant genomics [17].

CasΦ2 is a new Cas protein from huge phages. CasΦ2 is 789 amino acids and contains a C-terminal RuvC domain, and is about half the size of Cas9. CasΦ2 can process pre-crRNA into mature crRNA in the presence of Mg^2+^ irons. CasΦ2 has a wide PAM sequence (PAM: 5′-TBN-3′, B is G, T or C). It is confirmed that the genome editing function of CasΦ2 in vivo in plants is of great significance [7,18]. To verify the function of CRISPR/CasΦ2 system in plant genome editing, the model plants Arabidopsis and tobacco were selected; the genetic transformation of both is extremely easy. Agrobacterium-mediated transformation was used to obtain transgenic seedlings for gene editing efficiency analysis.

Here, we reported the application of CRISPR/CasΦ2 system in plant genome editing. *AtBRI1* and *NtPDS* of marker genes were successfully mutated, and mutant seedlings exhibiting the expected phenotype were observed. These results showed that a CRISPR/CasΦ2 system can be developed as a novel genome editing tool in plants. This system will benefit the research of plant functional genomics and, ultimately, crop breeding.

## 2. Results

### 2.1. CRISPR/CasΦ2 Vectors Construction

**Figure 1:**
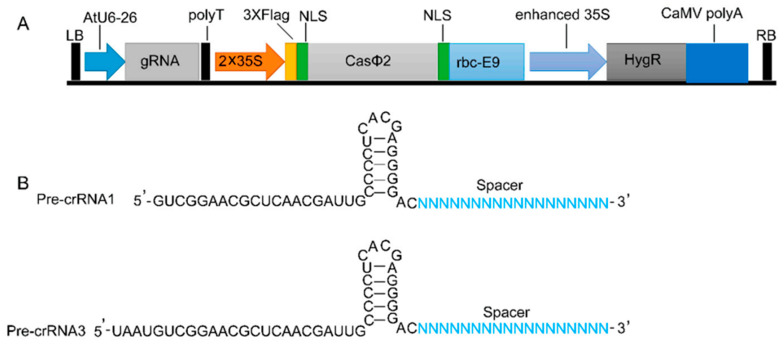
(**A**). T-DNA construct of CasΦ2 system for genome editing. (**B**). The structure of Pre-crRNAs.

To construct various vectors of the CRISPR/ CasΦ2 system, codon-optimized *CasΦ2* gene was cloned to replace the *SpCas9* gene in the binary vector pHSE401, resulting in 2 × 35S: CasΦ2 in which *CasΦ2* is under the control of the 2 × 35S promoter (Figure 1A). The Arabidopsis U6-26 promoter-driven sgRNA expression cassette can be cloned into T vector, creating different sgRNA type vectors (Figure 1B). 

### 2.2. Target Gene Vectors Construction

To verify the genome editing activity of CRISPR/CasΦ2 system in plants, the nuclease activity of CasΦ2 was tested targeting the endogenous *AtBRI1* and *AtPDS3* genes with two types of sgRNA. The loss of function of *AtBRI1* will generate male sterile and dark green plants with thickened leaves, etc. [19]. Loss of function of *AtPDS3* will generate albino phenotype [20]. The different sgRNA type targeted an 18-bp sequence in the Arabidopsis genes and tobacco gene, respectively. *AtPDS* gene was selected two target sites to construct target-transformation vectors, and *AtPDS* gene and *AtBRI1* gene were constructed two sgRNA type vectors of target site. *NtPDS* gene was constructed sgRNA1 type vector. Each sgRNA of the target gene was subcloned into pCasΦ2 vector by *Bsa* I site, the construction vectors of target genes used for transformation are described in Table 1. 

### 2.3. Analysis of Arabidopsis Plants Mutation

**Figure 2:**
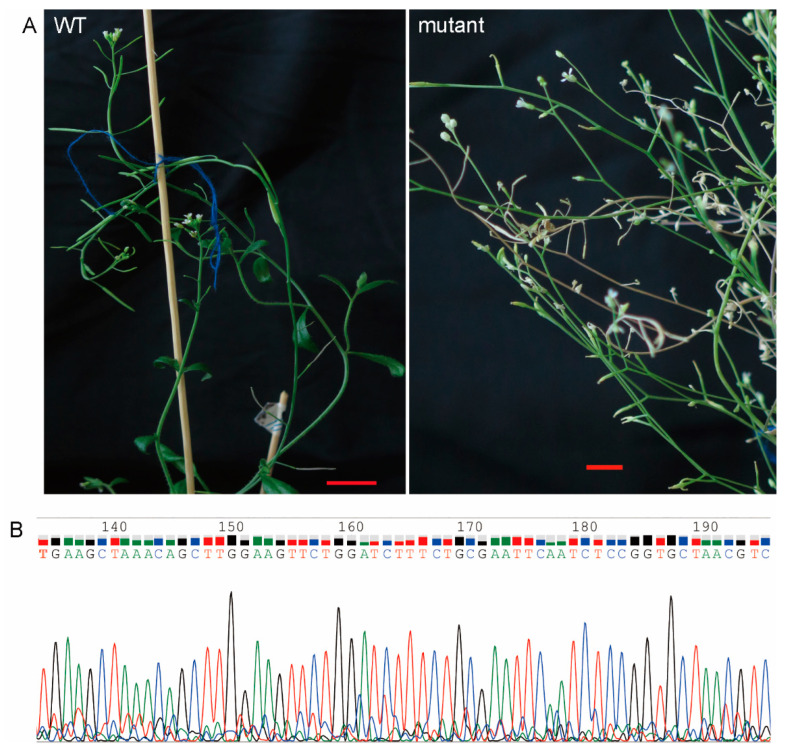
Generation of the Arabidopsis *AtBRI1* mutant. (**A**). *AtBRI1* mutant with phenotypes (right). (**B**). The mutation was detected by sequencing. WT: wild-type Arabidopsis. Bar = 1 cm.

The target vectors were transformed into Arabidopsis plants by *Agro*-*bacterium*-mediated transformation with infection solution containing 10 mM MgCl_2_. The harvested seeds were screened by 1/2 MS medium containing hygromycin to obtain resistant seedlings. Target regions in resistant lines were PCR-amplified and directly sequenced. For the AtPDS1 sgRNA1 type vector, we obtained 18 resistant lines, but no mutant lines were detected by sequencing. For the AtPDS1 sgRNA3 type vector, 17 resistant lines were no detected mutation by sequencing. sgRNA1 and sgRNA3 types of AtPDS2 vectors also found no mutations by sequencing. The AtBIR1 sgRNA1 type vector also did not detected the mutation by sequencing. However, mutation was detected in the resistant lines of the AtBIR1 sgRNA3 type vector by sequencing (Figure 2B). As the mutation line grew, we found expected mutant phenotype compared to wild-type plants (Figure 2A). For the AtBSD5 target editing of Cas9 system, we randomly selected four resistant lines by sequencing, two of which detected mutations. CasΦ2 is 789 amino acids, half the size of Cas9; CasΦ2 is 36 bp of sgRNA, and CasΦ2 may have more advantages than Cas9 in constructing multiple sgRNAs and editing multiple target sites. Together with sequence analysis of resistant lines, the results showed that the nuclease activity of CRISPR/CasΦ2 system was activated with Mg^2+^ irons, and sgRNA3 type guided CasΦ2 to edit target site sequence in plant cells.

### 2.4. Analysis of Tobacco Plants Mutation

**Figure 3:**
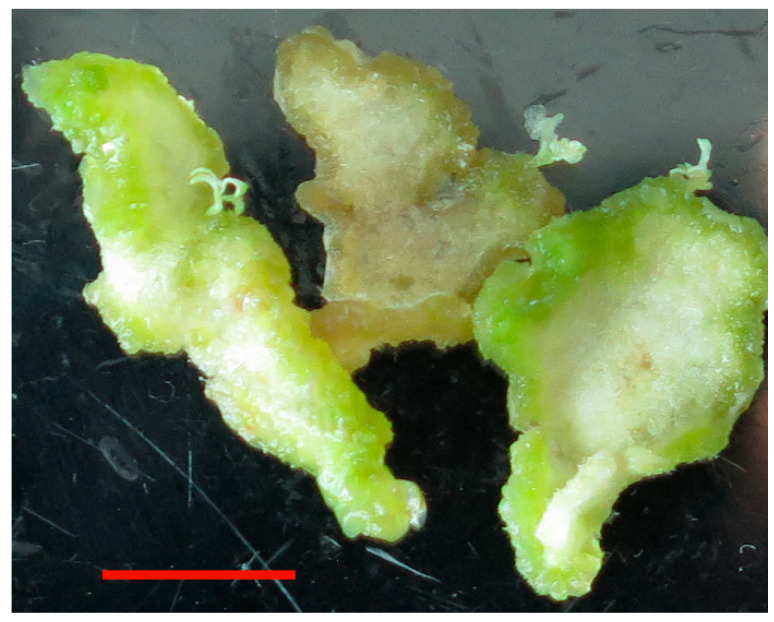
Transgenic plant regeneration of tobacco (*Nicotiana benthamiana*). Bar = 1 cm.

Next, we transformed NtPDS vector into tobacco leaves mediated by *Agrobacterium*, and we analyzed the mutation efficiency in transgenic tobacco by sequencing. When 10 nM MgCl_2_ was added to the medium, we found no albino seedlings. When 100 nM MgCl_2_ was added to the medium, we obtained 36 transgenic tobacco seedlings, 9 of which were albino seedlings (Figure 3). We sequenced the target site of albino seedlings, and mutations were detected. At a concentration of 1 mM MgCl_2_ in the medium, 27 transgenic tobacco seedlings were obtained, 8 of which were albino seedlings, and the mutations were verified by these lines. These results demonstrated that sgRNA1 type could guide CasΦ2 to edit target site sequence in the presence of Mg^2+^ irons.

## 3. Discussion

The CRISPR/Cas system is a simple, efficient and cheap genetic manipulation technology for genome editing in plants. At present, it is widely used in animals and plants genomics research and promotes our understanding of plant genetic function and improves crop traits. Crop varieties improved by CRISPR/Cas system were not easily distinguished from conventional-bred varieties, and these varieties were more resistant to abiotic and biotic stresses. This technique shortens the time for breeding and saves money and labor [21,22,23].

In our study, the CRISPR/CasΦ2 system has proved to be a useful and efficient tool for genome editing in plants. For targeting Arabidopsis *AtPDS* gene site vectors, resistant seedlings have no generated mutant lines by sequencing. We speculate that it may be due to the insufficiency Mg^2+^ ions, because target site sequence of CasΦ2 editing activity happened in the egg cells. For targeting Arabidopsis *AtBRI1* sgRNA1 type vector, no mutations have been detected by sequencing. The CRISPR/Cas system was used to edit target site at the egg cell stage in *Arabidopsis*; however, we used 35S promoter to drive *CasΦ2* gene expression. Moreover, the editing activity of CasΦ2 required Mg^2+^ to activate it. *AtBRI1* gene was edited by Cas9 system, and 68 transgenic plants were obtained from two different target sites, respectively. The expected phenotypes of these target sites were 48 and 33, respectively. We edited *AtBSD5* gene with Cas9, and we randomly sequenced four resistant plants and found two mutants. *Cas9* gene expression was driven by the egg cell-specific promoter compared with 35S promoter in *Arabidopsis*, and the efficiency of gene editing was improved [24,25]. However, by screening seeds infected with AtBRI1 vector of sgRNA3, two resistant seedlings were obtained, one of which had target site mutation and produced the expected phenotype. Using the CasΦ2 system in *Arabidopsis* editing, editing efficiencies were very low, mainly because, in the process of infection, there was a lack of Mg^2+^ ions. The editing function of CasΦ2 requires Mg^2+^ ions to be activated. Feng et al. used a CRISPR/Cas9 system to edit *PDS* gene, and only one expected albino plant was obtained in a large number of screenings. This happened because the expression cassettes of T-DNA were silenced [3]. The frequency of obtaining mutations may also be related to target genes and target site sequence [26]. Tobacco plants showed an expected albino phenotype, sequencing results verified that these plants had mutations because the mutation caused a frame shift during CDS frame. In tobacco transformation, the ability to generate mutant seedlings may be the reason for the presence of Mg^2+^ ions in the cultural medium.

At present, CRISPR technology includes CRISPR/Cas nucleases, base editors and prime editors. In particular, base editors and prime editors use dCas9 not to generate DSB to edit target site sequence [27]. Since CasΦ2 requires Mg^2+^ ions to activate gene editing activity, if Mg^2+^ ions are absent, CasΦ2 could be combined with base editor elements to edit directly at the target site sequence, and CasΦ2 would replace the nCas9 type base editing system. Moreover, CasΦ2 has 789 amino acids, half the size of Cas9, and CasΦ2 may have easier access to the nucleus in cells than Cas9 [18]. CasΦ2 has 36 bp of sgRNA, and CasΦ2 may have more advantages than Cas9 in constructing multiple sgRNAs and editing multiple target sites. There are off-target events in CRISPR/Cas9. The *ZmMS8* gene of maize was edited by Cas9, and the ms8 mutant line had off-target mutations, but it did not affect plant traits. The off-target events were eliminated by backcrossing or outcrossing [28].

## 4. Materials and Methods

### 4.1. Construction of the CRISPR/CasΦ2-Related Vectors

To construct CRISPR/CasΦ2 vector, the *CasΦ2* coding sequences from huge phages were codon-optimized for maize (*Zea mays* L.) and *Arabidopsis thaliana* and synthesized commercially (GenScript, Nanjing, China) [18]. The *CasΦ2* gene sequence included an N-terminal 3 × Flag and N- and C-terminal nuclear localization signals (NLSs) to ensure nuclear compartmentalization in plant cells, respectively. *CasΦ2* gene was directly cloned downstream of the 2 × 35S promoter cassettes from cauliflower mosaic virus (CaMV) by *Xba* I and *Sac* I sites from pHSE401 vector by replacing the *Cas9* gene [29]. Using the PCR cloning methods, a fragment containing two *Bsa* I sites was replaced the SpR fragment of pHSE401-CasΦ2 vector by *Hind* III site to produce the CRISPR/ CasΦ2 binary vectors, the fragment was amplified with the primer pairs HSBSF/R, PCR was performed using KOD-Plus-Neo (TOYOBO, Osaka, Japan), creating pCasΦ2 vector.

To construct sgRNA vector, AtU6-26 promoter sequence was amplified from *Arabidopsis* Columbia, the sgRNA and promoter sequences were amplified using overlapping PCR and the sgRNA sequence was cloned under the AtU6-26 promoter [29]. The sgRNA1 was amplified with the primer pairs U6P-F/gRNA1-R. The sgRNA3 was amplified with the primer pairs U6P-F/gRNA3-R. The PCR product was cloned into pEASY-Blunt Simple Cloning vector (Transgen, Beijing, China), creating sgRNA1T and sgRNA3T, respectively. Cas9 system as a control, we selected *AtBSD5* for target gene. All of the primers used in this work are listed in Supplementary Table S1.

### 4.2. Target Gene Vector Construction

For the target site construction of the target gene, the PAM 5′-TBN-N(18)-3′ (where B is G, T, or C) was chosen as the target site. To identify the PAM of CRISPR/CasΦ2 in plants, we constructed target gene vector for Agrobacterium-mediated plant transformation as follows. We selected *AtPDS* and *AtBRI1* gene of *Arabidopsis* and *NtPDS* gene of tobacco as the target genes. The *AtPDS* gene was selected two target site, one of which was target g_33 from Pausch et al. (AtPDS2 target site) [18]. To amplify promoter-sgRNA sequence, sgRNAT1 and sgRNA3T plasmid were used as template and amplified by primer pairs U6P-F/gRNA-target-R, respectively. The AtU6-26p termination sequence was amplified with another primers (target-F/U6T-R), the termination sequence product and promoter-sgRNA sequence product were then used as template to do overlapping PCR to generate sgRNA expression cassette with primers (U6P-F and U6T-R). Each sgRNA expression cassette was then inserted into the pCasΦ2 vector by *Bsa* I site, 60 ng pCasΦ2 vector and 15 ng desired sgRNA amplified fragment are combined with 35 U T4 DNA ligase (NEB), 10 U *Bsa* I (NEB), 1.5 µL 10 mM ATP and 1.5 µL 10 × CutSmart buffer (NEB) in a 15 µL reaction volume. The solution was incubated as follows: 37 °C for 5 min, 10 °C for 10 min, 20 °C for 5 min, final step 37 °C for 5 min, 15 cycles the conditions. Ten µL solution was transformed into Top10 chemically competent cells (Sangon Biotech, Shanghai, China) and transformations were sequenced.

To construct sgRNA of AtBSD5 of Cas9 system, the sgRNA was amplified with the primer pairs U6P-F/gRNA-BSD5-R, AtU6-26p termination sequence was amplified with another primers (gRNA-BSD5-F/U6T-R), sgRNA expression cassette was then inserted into the pHSE401 vector by *Bsa* I site.

### 4.3. Plant Growth and Plant Transformation

The seeds of Columbia-0 background were sterilized in ethanol for 30 s and 2.4% sodium hypochlorite solution for 5 min. The seeds were then rinsed 3 times with sterile water and were sown on 1/2 MS medium plates. After stratification at 4 °C for 2 d, the plates were transferred to a growth chamber at 21 °C with 16 h light/8 h dark. The construction vectors were transformed into *Agrobacterium tumefaciens* strain GV3101 by freeze–thaw method. *Arabidopsis* Col-0 wild-type plants were infected by floral dip method. The collected seeds were screened on 1/2 MS medium plates containing 50 mg/L hygromycin.

*Nicotiana benthamiana* plants were used for transformation. Plant were grown at 26 °C with 16 h light/8 h dark in the growth chamber. The target gene vectors were transformed into *Agrobacterium tumefaciens* strain GV3101 and used for Agrobacterium infiltration of tobacco leaves. Protocols for transfection and regeneration were the same as Baltes et al. [30]. The leaves were selected and regenerated to plantlets on MS medium containing 20 mg/L hygromycin, and co-culture medium and screening medium contained different concentrations of MgCl_2_ (10 nM, 100 nM and 1 mM MgCl_2_, respectively).

### 4.4. Target Gene Mutation Analysis

To identify the mutation in plants, genomic DNA was extracted from leaf tissues of transgenic seedlings using the Maxi CTAB method [31] and the genomic DNA was used as PCR template. Fragments surrounding the target sites were amplified with specific primers (Supplementary Table S1), a region from 524 bp to 800 bp including the target site of target gene was amplified by PCR. The fragment of *AtBRI1* gene was amplify with two pairs of primers AtBRI1-F/R. The target AtPDS1 and AtPDS2 fragment of *AtPDS* gene were amplify with two pairs of primers AtPDS1-F/R and AtPDS2-F/R, respectively. The fragment of *AtBSD5* gene was amplify with two pairs of primers AtBSD5-F/R. The fragment of *NtPDS* gene was amplify with two pairs of primers NtPDS-F/R. PCR protocol was as the following conditions: 36 cycles of 1 min at 94 °C, 30 s at 60 °C, and 1 min at 72 °C, final step 5min at 72 °C. The PCR products were then analyzed by agarose gel, and the PCR products were sequenced to identify mutations. All of the primers are listed in Supplementary Table S1.

## Figures and Tables

**Table 1:** The target gene vectors and sgRNA target sites used in this study.

Target Gene Vector	Plant	Gene Target	sgRNA Type	Target Site (5′-3′)
AtPDS1-1	*Arabidopsis*	*AtPDS3*	sgRNA1	CACCAGCAGAGGAATGGA
AtPDS1-2			sgRNA3	
AtPDS2-1			sgRNA1	CAGTTGACAATCCAGCCA
AtPDS2-2			sgRNA3	
AtBRI1-1		*AtBRI1*	sgRNA1	CTGCGAATTCAATCTCCG
AtBRI1-2			sgRNA3	
NtPDS	*Nicotiana benthamiana*	*NtPDS*	sgRNA1	GTAGTAGCGACTCCATGG

## Data Availability

Not applicable.

## Supplementary Materials

The following supporting information can be downloaded at: https://www.mdpi.com/article/10.3390/ijms23105755/s1.
